# The DNA-binding network of *Mycobacterium tuberculosis*

**DOI:** 10.1038/ncomms6829

**Published:** 2015-01-12

**Authors:** Kyle J. Minch, Tige R. Rustad, Eliza J. R. Peterson, Jessica Winkler, David J. Reiss, Shuyi Ma, Mark Hickey, William Brabant, Bob Morrison, Serdar Turkarslan, Chris Mawhinney, James E. Galagan, Nathan D. Price, Nitin S. Baliga, David R. Sherman

**Affiliations:** 1 Seattle Biomedical Research Institute, Seattle, Washington 98109, USA; 2 Interdisciplinary Program of Pathobiology, Department of Global Health, University of Washington, Seattle, Washington 98195, USA; 3 Institute for Systems Biology, 401 Terry Avenue North, Seattle, Washington 98109, USA; 4 Department of Chemical and Biomolecular Engineering, University of Illinois Urbana-Champaign, Urbana, Illinois 61801, USA; 5 Department of Biomedical Engineering, Boston University, Boston, Massachusetts 02215, USA; 6 Department of Microbiology, Boston University, Boston, Massachusetts 02215, USA; 7 Bioinformatics Program, Boston University, Boston, Massachusetts 02215, USA; 8 The Eli and Edythe L. Broad Institute of Harvard and MIT, Cambridge, Massachusetts 02142, USA

## Abstract

*Mycobacterium tuberculosis* (MTB) infects 30% of all humans and kills someone every 20–30 s. Here we report genome-wide binding for ~80% of all predicted MTB transcription factors (TFs), and assayed global expression following induction of each TF. The MTB DNA-binding network consists of ~16,000 binding events from 154 TFs. We identify >50 TF-DNA consensus motifs and >1,150 promoter-binding events directly associated with proximal gene regulation. An additional ~4,200 binding events are in promoter windows and represent strong candidates for direct transcriptional regulation under appropriate environmental conditions. However, we also identify >10,000 ‘dormant’ DNA-binding events that cannot be linked directly with proximal transcriptional control, suggesting that widespread DNA binding may be a common feature that should be considered when developing global models of coordinated gene expression.

## Abstract (web summary)

Adaptation of *Mycobacterium tuberculosis* to the host environment is principally mediated through its transcription factors. Here, the authors report the DNA binding and transcriptional profile of ~80% of all predicted *M. tuberculosis* transcription factors, and find wide-spread dormant DNA binding.

## Graphical abstract

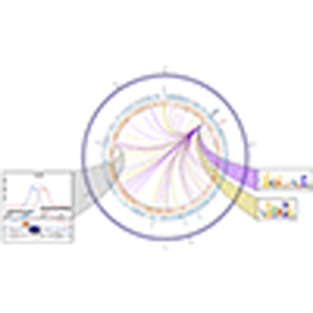

M*ycobacterium tuberculosis* (MTB) is a remarkably successful pathogen that infects an estimated 1.5 billion people and kills 1.3 million people each year^1^. Throughout TB disease, both bacterium and host engage in a dynamic series of adaptations to modulate local environments. For the pathogen, adaptation is principally mediated through the ~214-DNA-binding proteins encoded in the MTB genome. These proteins interact with small molecule chemical messengers, other proteins and the DNA to shape the transcriptional landscape of the cell and convert cascading stimuli into coordinated effector gene responses. Several approaches to understand the wiring and connectivity of interacting macromolecular components of MTB have been described, including gene expression pattern-driven identification of regulatory subnetworks^2^,^3^, metabolic reconstructions^4^,^5^, integration of expression data from diverse experimental and environmental conditions^6^ and hybrid networks that seek to bridge transcription regulation with metabolic outputs and cellular fitness^7^. In each case the goal of these approaches is to constrain the universe of potential interactions within cells through an iterative process of experimentation, data collection and computational approaches that result in network reconstruction.

Various groups have probed the gene regulatory landscape of MTB by characterizing the regulons of individual transcription factors (TFs). The most widely applied approach has been gene knockout and phenotyping or transcriptional profiling of the resultant mutant^8^,^9^,^10^. More recently, however, technologies such as chromatin immunoprecipitation followed by microarray hybridization or high-throughput sequencing (ChIP-chip and ChIP-seq, respectively) have been applied to MTB^11^,^12^,^13^,^14^,^15^,^16^. These approaches identify directly sites of TF-DNA binding, and in conjunction with transcriptional profiling and/or meta-analyses offer a powerful window in to the global regulatory capacity of individual proteins. Employing ChIP-seq and transcriptional profiling, we recently described an analysis of the binding profile for 50 MTB TFs assessed in a uniform condition^17^. This preliminary network reconstruction showed good concordance with published results, as well as common features of regulatory networks from other organisms, such as robust network construction, connectivity and DNA-binding motif structure^18^,^19^,^20^.

Here we expand efforts to characterize the MTB gene regulatory network. We report the DNA binding and transcriptional regulatory profile of ~80% of all predicted MTB TFs (>150 proteins). From these data we derive high-confidence DNA consensus motifs for >50 TFs. We show that chromosomal regions proximal to coding sequence or transcription start sites are enriched for binding, allowing us to define functionally a genome-wide promoter window size for MTB. We identify 5,400 protein–DNA interactions within this window with high probability for direct transcriptional control of proximal targets, and 1,162 binding events that regulate proximal gene expression in the experimental condition assayed. However, we also note even more DNA binding that cannot be linked directly with transcriptional control. Further, we characterize one TF in which widespread binding events, most of which are not directly associated with gene expression changes, are nonetheless dictated by specific DNA sequence motifs that can be validated by an independent experimental approach. We propose the phrase ‘dormant binding’ to describe sequence-specific protein–DNA interactions without a proximal effect on gene expression, and suggest that this class of binding may exert proximal regulatory control under different environmental conditions, but may also contribute more subtly to the regulatory landscape of the cell. Altogether, this work presents an experimentally constrained protein–DNA interaction framework for MTB that reveals thousands of DNA-binding events, many of which we can link to proximal regulatory events. Our pan-genome survey indicates that widespread, dormant TF-DNA binding is very common, and suggests that the control of gene expression in bacteria may involve a layer of complexity that is currently unappreciated.

## Results

We recently described a preliminary MTB gene regulatory network based on the DNA-binding patterns of 50 TFs (23% of the 214 TFs of MTB)^17^. Here we present a substantially more complete transcriptional regulatory network that incorporates updated peak calling algorithms, stringent controls/filters to define high-quality TF-binding (see Methods), and includes 80% of the MTB TFs (workflow in Supplementary Fig. 1).

We cloned 206 (of the estimated 214) DNA-binding genes into an anhydrotetracycline-inducible Gateway shuttle vector to contain an N- or C-terminal FLAG epitope tag. The remaining eight genes proved refractory to our subcloning efforts. For added inclusiveness, this list was compiled through gene annotation data from Tuberculist^21^, TBDB^22^ and PATRIC^23^, as well as manual curation^24^. Once transformed, we cultured MTB strains to a uniform growth stage and induced expression of the gene-of-interest for 18 h—approximately one cell division. We then harvested chromatin samples for ChIP-seq as well as total RNA for high-density transcriptional profiling by custom tiled microarray. For microarray analysis, induction and experiments were repeated with at least three biological replicates^24^. For ChIP-seq samples we employed a custom algorithm for read alignment and ChIP peak calling (Methods).

### ChIP-seq data set and controls

Previously, we showed that DNA-binding events reproduced with high fidelity in eight of eight replicate ChIP samples^17^. In addition to the experimental ChIP samples we created a negative control composite data set against which we filtered experimental ChIP data sets. Because no single control captures all known or potential ChIP artifacts we designed this negative control compendium to include 10 diverse samples/sequencing data sets: wild-type (WT) H37Rv chromatin immunoprecipitated with and without anti-FLAG antibody (input DNA and mock immunoprecipitation (IP) controls, respectively), chromatin samples from uninduced expression-vector-bearing cells immunopreciptated with and without anti-FLAG antibody (basal expression from chimeric inducible promoter and mock IP controls, respectively), as well as chromatin samples from induced non-TF genes immunoprecipitated with anti-FLAG antibody (specificity of FLAG IP). We subjected each control data set to peak calling, creating an experimentally derived negative control peak set consisting of ~2,000 scored final peaks. We then compared each experimental peak with this negative control peak set to define a collection of pass-filter DNA-binding events (Methods). This approach identified both global and local binding patterns for every TF assayed with associated significance scores for every ChIP peak (Supplementary Fig. 2).

Some genomic regions appeared to be hotspots for ChIP enrichment, irrespective of the significance threshold. Recent reports from yeast^25^,^26^ suggest that loci with high transcriptional activity can be artificially enriched in ChIP assays. We compared our MTB high-occupancy sites against the absolute log_2_ expression value of transcripts derived from more than 700 microarrays^24^ and did not observe any such correlation with hyperenriched regions and transcript abundance (Supplementary Fig. 3). Nevertheless, we know of no biological mechanism for why these loci should be enriched across TF class and experiment. Therefore, any 50-bp region bound by more than 50 different TFs was flagged as a provisional experimental artifact and removed from subsequent analysis. This step culled 1,006 peaks at five gene loci (Rv1088, Rv1115, Rv1396c, Rv2190c and Rv3622c-3623).

We considered the possibility that artificially high TF gene induction from our ectopic expression system might result in more DNA binding than would be observed in WT cells. In addressing that question, we previously demonstrated good concordance between DNA binding following ectopic induction of tagged TFs and published genome-wide binding studies that relied on native conditions and/or antibodies^12^,^14^,^17^,^22^. Specifically, we compared data from our overexpression system to results of ChIP-seq experiments using WT cells and antibodies directed at native BlaI^12^, DosR^11^ or EspR^14^. In each case, approximately the same number of peaks was identified, and peak position and height were well conserved^17^. To assess this question more broadly, we compared here the number of binding sites per TF and the magnitude of TF ectopic induction, and found no correlation (Supplementary Fig. 4a). In addition, we compared TF expression levels in our overexpression system to a compendium of >2,300 published microarrays. We found that >80% of TFs were induced to a higher level by one or more experimental condition in WT cells (Supplementary Fig. 4b and ref. 24). Thus, while we cannot exclude the possibility that overexpression sometimes produced nonphysiological DNA binding, we conclude that such spurious DNA associations are rare in our data sets.

### Network topology and characteristics

We analysed genome-wide binding profiles for all TFs at *P* value cutoffs of <0.05, <0.01, <0.001 and <0.0001. As expected, the number of protein–DNA interactions shrinks as we progress to more stringent inclusion thresholds (Supplementary Fig. 5). While binding events in the range 0.05>*P*>0.01 have binding scores stronger than at least 95% of all negative control peaks and are clearly distinguishable from background, they generally possess lower signal-to-noise ratios and skewed read distributions (Supplementary Fig. 2). Testing showed that DNA-binding events with a *P*~0.01 could be confirmed by independent experiment, where peaks with *P*~0.05 were less consistently validated. We therefore chose a cutoff of *P*<0.01 to filter peaks for subsequent analyses. With this threshold the physical DNA-binding map includes 15,980 protein–DNA interactions from 156 MTB TFs. Supplementary Table 1 provides all TF–target interactions, with associated peak-binding metrics, genome coordinates and confidence scores. In addition, all raw and filtered data can be found at: http://networks.systemsbiology.net/mtb.

**Figure 1:**
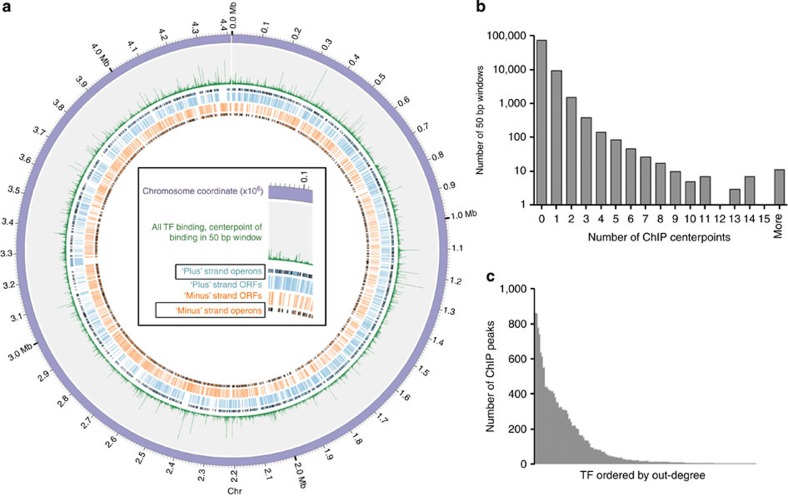
A global view of DNA binding. (**a**) TF-binding sites identified by ChIP-seq plotted with Circos^59^. Sense (blue) and antisense (orange) CDS and operon boundaries illustrated with black edges. The 4.4-Mb H37Rv chromosome is divided into nonoverlapping 50-bp windows, and green spikes represent the total number of TF-binding events within each window. (**b**) Histogram of number of TF-binding events per 50-bp window. (**c**) Number of ChIP-binding events (out-degree) for each of the 156 DNA-binding proteins with at least one binding site.

We mapped the centre of each binding event peak, and the global distribution of all TF-binding events was visualized on a circularized map of the MTB chromosome (Fig. 1a). Although thousands of genome-binding events were mapped, visualization at high resolution revealed that the chromosome in general is sparsely bound (Fig. 1b). The vast majority of the genome (~3.8 million base pairs, 86%) was not associated with any TF binding, whereas ~0.6 million base pairs contained at least one binding site, and locations with more binding events were progressively fewer. Regions with multiple TFs binding in close proximity are prime candidates for combinatorial regulation. For example, our data recover the well-characterized binding of Rv3133c/DosR upstream of both Rv3134c and Rv2031c^8^,^11^; however, we also note in both regions a strong binding signature from hypoxic-responsive TF Rv1985c^27^.

For individual TFs in this study, the number of DNA-binding events per protein ranged from 0 to >850 (Fig. 1c). No binding sites were detected for 24 TFs. There were also seven proteins with >500 binding sites each on the chromosome, and 14 TFs accounted for ~50% of all binding in the network. For proteins that do not bind DNA as well as for prolific binders, no single gene family describes these TFs.

### Correlating DNA binding with the regulation of transcription

We explored binding locations relative to translation start sites of annotated genes. About 25% (nearly 4,000 out of ~16,000 binding sites) were within intergenic regions. While this is roughly 2.5 × what would be observed by random chance (cumulative hypergeometric mean *P*<0.001), the relatively low 25% intergenic enrichment was unexpected and caused us to investigate further binding site distribution characteristics. Peaks with the highest quality scores were slightly more likely to be intergenic. For instance, among the 800 best peaks, the proportion within intergenic regions rose to 29% (Supplementary Fig. 6). However, even when considering only the highest scoring peaks (top 20%) on a per-TF basis, the binding site distribution is highly idiosyncratic (Supplementary Table 2). About one-fourth of TFs exhibit 80% intergenic binding or more, while another one-fourth show at least 80% binding within coding sequences. Of the proteins with strong intergenic bias in this analysis, nearly all bind three or fewer times on the MTB chromosome. We cannot exclude the possibility that the prevalence of within-gene DNA binding we report is somehow a function of our approach; however, the trends observed here are broadly consistent with other genome-wide DNA binding studies in MTB^14^,^16^, and with some reports in other bacteria^28^,^29^.

**Figure 2:**
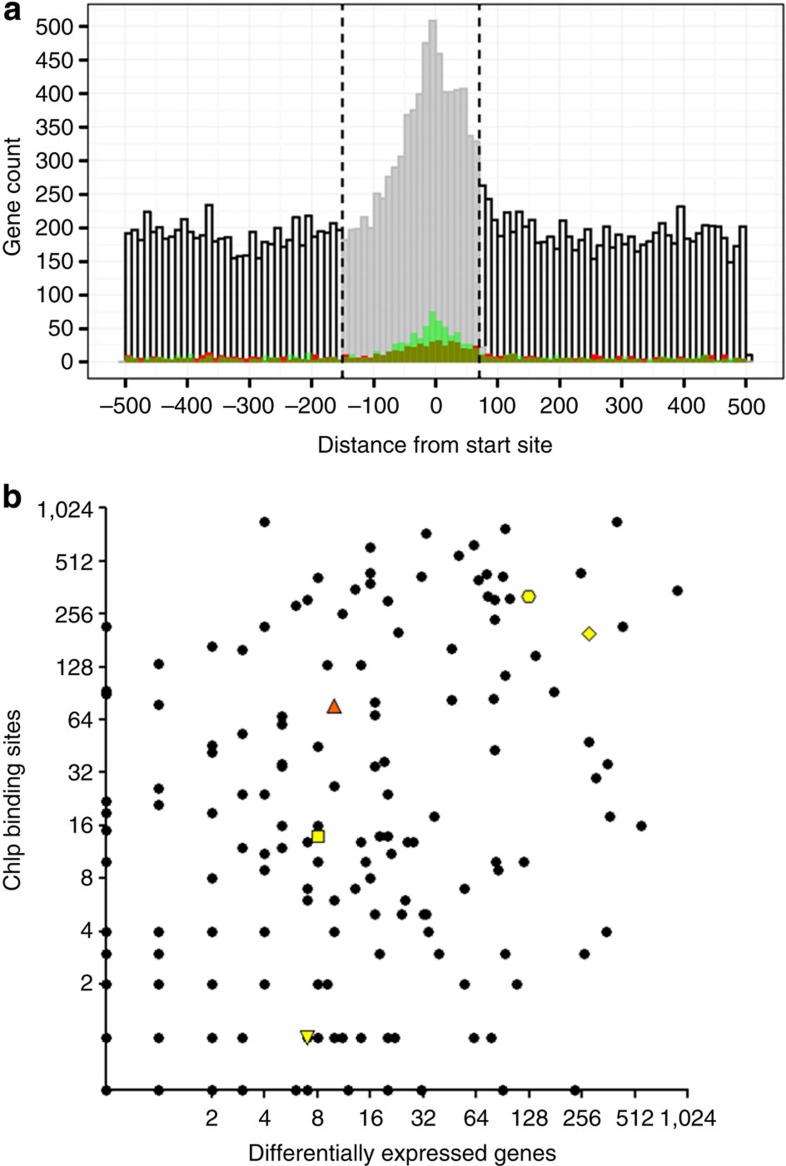
Global view of DNA-binding and transcriptional regulation in MTB. (**a**) Plot of binding distribution in the 1-kb nucleotide window (−500 to +500) surrounding CDS or transcription start sites. ROC/AUC analysis indicated that the optimal promoter window size is −150 to +70 nucleotides (indicated by vertical dashed lines and shading of histogram). Binding events correlated with 1.5-fold induction or repression in the overexpression data set^24^ are depicted in red and green, respectively. (**b**) The relationship between the number of binding events detected versus the number of transcriptional changes associated with induction of each TF in this study. Rv0494 (orange triangle) is an example of a TF with prolific binding but limited differential gene expression. Additional genes discussed in text highlighted in yellow with symbols as follows: Rv1657/ArgR (downward triangle), Rv1846v/BlaI (square), Rv3133c/DosR (octagon), and Rv3849/EspR (diamond).

We also analysed the binding locations relative to an experimentally determined map of transcriptional start sites (TSS) in MTB, many of which are not consistent with traditionally defined coding region boundaries^30^. We observed a striking enrichment of TF-binding proximal to TSSs, with the highest density of binding at −18 nucleotides upstream to TSSs (Fig. 2a). To associate TFs with direct regulation of target genes, we analysed instances where TF overexpression resulted in significantly altered expression of genes proximal to TF-binding locations (Methods and ref. 24). By performing this analysis over different sized genomic segments, we determined that a consensus promoter spanning 150 bp upstream to 70 bp downstream of starts yielded maximal sensitivity versus specificity (Supplementary Fig. 7). All binding events within this window were considered functional, that is, capable of directly regulating downstream gene expression in the right environmental context. In all, 5,400 binding sites for 143 TFs were located within promoter windows. Because a single binding site could be associated with more than one promoter, altogether there were 7,248 TF–promoter interactions within 2,848 promoters. There were 1,243 promoters with a single TF-binding site, and the median was two binding sites per promoter. Overexpression of TFs under reference growth conditions validated that 1,162 TF–DNA interactions can directly regulate proximal genes (Supplementary Table 3). Thus, despite the known conditional nature of gene regulation, we were able to validate over 20% of all promoter-proximal binding events using only one reference laboratory growth condition. By extension, a large fraction of the >7,200 promoter-proximal TF–DNA interactions are likely to regulate gene expression directly in the appropriate environmental context, and can even be used to refine promoter predictions. For example, expression of the putative benzoquinone methlytransferase Rv0560c was previously predicted to be controlled by an unknown repressor of the MarR family^31^. We found five TFs that bind near the start of this gene, but of those only overexpression of the MarR family TF Rv2887 resulted in the repression of Rv0560c (Supplementary Fig. 8). However, the other TFs are strong candidates to regulate Rv0560c in other contexts. Mapping TF–DNA binding and expression changes in other environments should expand further the list of interactions with corresponding identifiable downstream expression changes^6^.

While 5,400 DNA-binding events are located in the promoter window, roughly 66% (>10,500) of binding sites are outside this region. Altogether, 109 different TFs exhibit promoter-distal binding. While there are examples of prokaryotic proteins binding outside of promoters and exerting regulatory effect at a distance (Fig. 2a and refs 32, 33, 34, 35), as a class these binding events are less likely to exert direct influence on gene expression. To explore globally the link between TF–DNA binding and transcription, we compared the number of binding events per TF and the number of expression changes associated with each TF (Fig. 2b). Of 178 MTB TFs in this study, nearly 40% exhibit an approximately linear relationship between the number of DNA-binding events and transcriptional changes. Two of the most well-characterized DNA-binding proteins in MTB (Rv3133c/DosR^8^ and Rv3849/EspR^14^) behave this way. For roughly 30% of proteins, induction is associated with a disproportionately large impact on transcription relative to the number of binding sites. These proteins may regulate other TFs and initiate a transcriptional cascade. Alternatively, some of these TFs may be poor candidates for ChIP analysis. In contrast, there are approximately the same number of proteins whose induction results in prolific DNA binding but comparatively few transcriptional changes. The regulatory circuits of these genes may be complex, perhaps requiring one or more partner TF(s) or another cofactor to reconcile DNA binding and expression profiles. These proteins belong to a wide range of TF families, including TetR, ArsR and GntR, along with one nucleoid-associated protein Lsr2.

### Identifying DNA consensus motifs from ChIP-seq data

We searched for conserved motif signatures for each TF. We queried all DNA-binding data using MEME^36^ and default parameters. We performed each motif search twice for each grouping—one unconstrained and one constrained—to detect only palindromes. After filtering motifs for MEME *E*-values (*E*<=1) and peak locations within the queried sequence (*P*<=0.05) we could identify significant motifs for a total of 57 (71%) out of the 80 TFs that had ≥14 ChIP-seq peaks. We report the two motifs detected for each TF, along with all related statistics, in Supplementary Table 4. TFs with a greater number of binding sites were more likely to have an identified consensus motif. The average number of binding sites for TFs with a motif was 246 (range 14–859), compared with an average of 28 peaks (range 3–437) for those TFs where a significant consensus motif could not be identified. For TFs with previously characterized DNA-binding motifs, this analysis corresponded well with previous reports (for example, Rv2506 (refs 37, 38), Rv2359 (ref. 39), DosR (ref. 8), KstR (ref. 9) and EspR (ref. 14)). In cases where the data set was of sufficient size to parse by location within or outside of a promoter, the identified consensus motifs tend to share the dominant sequence features of the motif derived from the aggregate sequences (for example, Rv1255c); however, in this context subtle sequence variations are likely to have functional consequences.

### Rv0494 as an example of widespread binding

**Figure 3:**
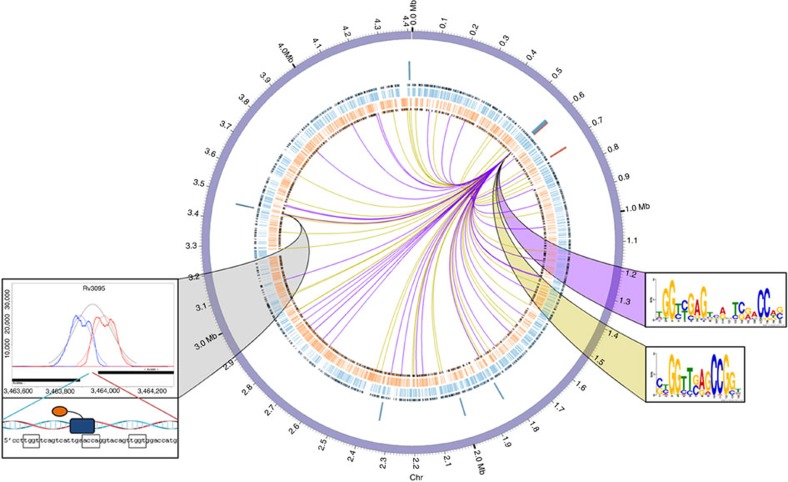
Rv0494 as an example of widespread binding and local gene regulation. Rv0494 binding and regulation of gene expression was plotted using Circos^59^, with sense (blue) and antisense (orange) CDS and operon boundaries illustrated with black edges. The Rv0494 gene locus is positioned at ~2 o’clock, and ChIP-binding sites are denoted at the terminus of each edge radiating out from Rv0494 (peaks of *P*<0.001 indicated by purple lines, 0.001<*P*<0.01 indicated by yellow lines). Rv0494 consensus motifs corresponding to peak significance thresholds are indicated by the colour-matched ribbons. Genes that exhibit significant differential regulation (more than twofold change with empirical Bayes method *P*<0.01 from five biological replicate microarrays) upon induction of Rv0494 are indicated by blue (gene repression) and red (gene induction) bars. The Rv0494 ChIP-binding site between regulated genes Rv3094c–Rv3095 is shown connected by a grey ribbon.

As indicated above, ~30% of the TFs in this study bind prolifically around the chromosome both within and outside of promoters, but affect relatively few transcriptional changes. To investigate this behaviour, we focused on a representative member, Rv0494 (Fig. 3). Rv0494 is a GntR-family regulator^40^,^41^ whose induction correlated with 10 transcriptional changes at seven genomic loci (Fig. 3, blue–red ring) including binding at the Rv3094c–Rv3095 locus (Fig. 3, grey ribbon); however, there are 77 Rv0494-binding events distributed around the MTB chromosome (Fig. 3—internal lines). DNA pattern searching using MEME^36^ on the entire data set yielded two significant consensus motifs (Supplementary Table 4). We observed that the Rv0494-bound regions contributing to the longer (17mer) motif have more significant ChIP-binding scores, whereas the bound regions contributing to the shorter (~9mer) motif have strong but less significant scores. We stratified ChIP-binding sites by score and searched for consensus motifs in two tranches: *P*<0.001 (higher peak quality scores; 36 input regions, purple lines in Fig. 3) and 0.001<*P*<0.01 (lower peak quality scores; 41 regions, yellow lines in Fig. 3). We saw a striking division in the consensus motifs derived. Of the 36 highly significant binding sites, 35 contained a close variant of the 17-mer consensus motif (motif *E*-value=8.4 × 10^−51^, Fig. 3, purple ribbon). Of the 41 less significant bound sequences, 28 contained the 9-mer consensus motif (motif *E*-value=1.7 × 10^−31^, Fig. 3, yellow ribbon). Combining the bound regions that did not contribute to either motif initially, we found that these peaks had *P* values in the middle of the distribution (0.0015<*P*<0.004, Supplementary Fig. 9). Repeating the MEME pattern search on these 14 regions showed that 13 sites contained a close variant of the 17-mer consensus motif (motif *E*-value=8.3 × 10^−5^).

We next analysed expression from Rv0494-induced cultures. Of the 10 differentially expressed genes following Rv0494 induction, two of these loci (six genes) are immediately adjacent to Rv0494-binding sites. These are strong candidates for direct regulation by Rv0494, and both these loci show highly significant binding (Fig. 3, grey ribbon, and Supplementary Table 3). However, we also find examples of binding to the strong consensus motif with no obviously associated change in gene expression.

### Validating Rv0494 binding to different motifs

**Figure 4:**
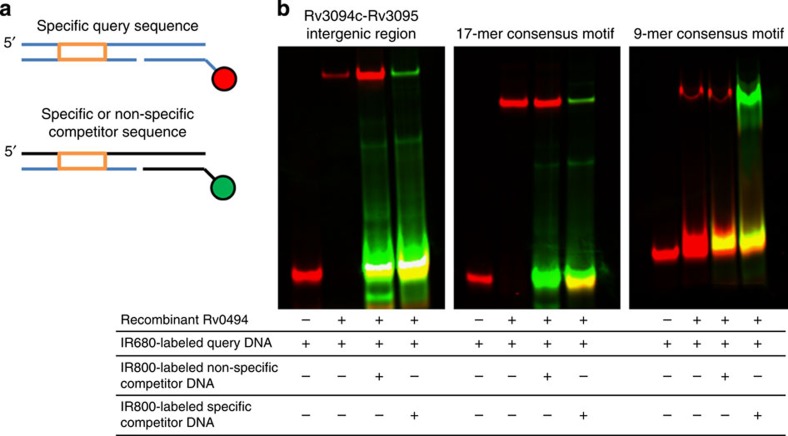
Independent validation of Rv0494 binding. (**a**) Schematic of DNAs used in these experiments. Three DNAs are annealed to form a single dsDNA product: a specific query sequence (orange box) is annealed in a 3-piece dsDNA fragment to a unique 12-mer sequence covalently coupled to a reporter dye. In these experiments, the specific query DNA was labelled with IR680 (red) and specific or nonspecific competitor DNAs were labelled with IR800 (green). (**b**) Purified recombinant Rv0494 binds specifically to ChIP-identified wild-type sequence (left panel), the 17-mer consensus motif (middle panel) and the 9-mer consensus motif (right panel). In the absence of protein, dye-coupled DNA does not shift (lane 1); however, the protein–DNA complex runs at a higher molecular weight (lane 2). This protein–DNA complex persists in the face of 20 × molar excess green-labelled nonspecific competitor DNA (lane 3), but can be outcompeted by the addition of 20 × molar excess green-labelled specific competitor DNA (lane 4).

From these analyses, the vast majority of Rv0494-binding sites—76 of 77 bound regions—are described by one of two consensus motifs. We sought to validate this binding by an alternate approach. Employing purified, recombinant Rv0494 protein we developed a ‘universal’ electrophoretic mobility shift assay (uEMSA) in which a uniform DNA scaffold was modified to contain a 5′ IR680 (red) or IR800 (green) IR tag (Fig. 4a). This approach allows simultaneous visualization of target, nonspecific and specific competitor DNAs in an *in vitro* EMSA^42^. The Rv3094c–Rv3095 intergenic region contains a variant of the 17-mer motif (Fig. 3), and in uEMSA experiments both the 17-mer consensus motif and Rv3094c–Rv3095 DNA sequences are tightly bound by recombinant Rv0494 protein (Fig. 4b). Binding is specific, as confirmed by a persistent gel shift in the face of 20 × molar excess nonspecific competitor DNA; however, in the face of 20 × molar excess-specific competitor, the Rv0494 protein preferentially binds to the more abundant IR800-labelled competitor DNA. The Rv0494 protein also showed specific binding to the 9-mer DNA consensus motif, although at a higher protein concentration than the 17-mer motif. We note that none of the 9-mer-Rv0494 interactions were associated with detectable changes in proximal gene expression, indicating that such binding events can nonetheless be validated by alternate means. Altogether, these data indicate that consensus DNA-binding motifs derived from ChIP-seq can be validated by alternate experimental methods, and demonstrate a correlation between ChIP peak quality score and protein–DNA affinity.

## Discussion

Robert Koch described the cause of tuberculosis more than a century ago yet MTB remains a pervasive pathogen, infecting 30% of the world’s population and causing two to three deaths every minute. To understand better how MTB adapts within the human host we undertook a systematic characterization of the gene regulatory network. We ectopically induced expression of epitope-tagged copies of nearly every DNA-binding protein in MTB (Supplementary Fig. 1). Using this approach we performed ChIP-seq and transcriptional profiling under a uniform condition for 178 TFs. We filtered the binding patterns of experimental samples against a robust negative control peak set and imposed a stringent significance threshold for inclusion of DNA-binding events in downstream analyses. We also associated binding with gene expression changes, incorporating transcriptional data generated under the same experimental conditions. These data provide an in-depth, system-wide view of the DNA-binding network in this important bacterial pathogen.

The MTB DNA-binding network consists of ~16,000 protein–DNA interactions from 154 genes that passed our stringent filter set (Fig. 1). We could not identify consistent attributes to define the 24 proteins that did not bind DNA, and we hypothesize that these proteins require additional signals or modifications to bind the chromosome. We also noted prolific binders—seven proteins with >500 binding sites each. MEME pattern searching analysis revealed significant consensus motifs for each of these proteins, which suggests that prolific binding was still dictated by sequence-specific DNA interactions (Supplementary Table 4). The number of binding events per protein could be fit to a power law distribution (*p*(*k*)~*k*^−1.5^), with half of the binding coming from 14 proteins and ~90% of the binding from 44 proteins (~25% of all assayed binding proteins, Fig. 1c). However, from the perspective of the DNA the chromosome is sparsely bound. More than 85% of the genome bound no TFs, while slightly more than 10% of the genome bound a single TF (Fig. 1b). A few loci (~2.5%) were hotspots for binding, and these are prime candidates for combinatorial protein–DNA interactions.

Genome-wide, TF binding was nonrandom, and we identified significant consensus motifs for 57 TFs (Supplementary Table 4). Furthermore, we observed more than twice as much binding in intergenic regions than would be expected by random chance. Similarly, we found a striking enrichment of TF binding within −150 to +70 nucleotides of annotated start sites (CDS or TSS), with the greatest enrichment in the 0 to −20 region. Altogether, we found approximately one-third (5,400 of 15,980) of TF-binding sites were within one or more 220-bp promoter windows, resulting in >7,200 TF–promoter interactions (Fig. 2a and Supplementary Table 3). More than 1,150 of these binding events were associated with altered gene expression in our experiments, and in the appropriate environmental context, many more of these >7,200 interactions are likely to serve a proximal regulatory function. However, even more binding events (>10,500) were positioned outside of promoters. We observe some instances of promoter-distal binding correlated with proximal gene regulation (Fig. 2a), and probably in alternate environmental contexts a greater number of these would act to alter expression of proximal genes. However, it is also likely that many of these promoter-distal binding sites are transcriptionally dormant. Abundant promoter distal binding has been noted before^32^,^33^,^43^, and in some cases individual proteins that bind DNA prolifically have been shown to regulate transcription at a subset of their loci but not at others^14^,^16^,^34^. For instance, in MTB the TF EspR has been labelled both a specific transcription factor^44^ and a nucleoid-associated protein^14^. Our analysis provides evidence for both ideas. We find that EspR exhibits binding that is both widespread and promoter-proximal, and that only a fraction of binding events directly influence transcription. Furthermore, we observe similar behaviour from the majority of TFs in MTB.

To examine further the phenomenon of widespread binding with limited regulation we focused on Rv0494, which binds 77 times and promotes altered expression at only two of these loci (Fig. 3). We identified consensus motifs associated with both stronger and weaker binding and protein–DNA interactions could be validated by independent experimental approaches (Fig. 4). Some Rv0494-dependent expression changes were proximal to strong binding events; however, many strong binding events were not associated with any local gene expression changes.

Altogether, our analyses both complement and contrast with current models of bacterial transcription. For example, we found numerous strong DNA-binding consensus motifs (Supplementary Table 4) and robust enrichment for DNA binding in the window (−150 to +70) relative to transcription start sites (Fig. 2a), in agreement with promoter studies in bacteria^45^. However, compared with the model bacterium *Escherichia coli*, the MTB TF-DNA-binding network results were surprising in terms of binding site numbers, locations and effects. Transcription is well studied in *E. coli*, with substantial information collected and curated at the online repository RegulonDB^46^. This database lists ~2,400 *E. coli* TF-DNA-binding events, nearly 7 × fewer than we observe in MTB. Only 27 individual *E. coli* TFs are known to bind DNA more than 20 times, compared with 69 in MTB. Further, in MTB we find dozens of TFs with widespread binding and few downstream transcriptional changes.

How to reconcile these differences? We have considered the possibility that widespread DNA binding is an artifact of the ChIP approach. However, we have ruled out previously described artifacts such as spurious ChIP enrichment proximal to highly transcribed loci (Supplementary Fig. 3), applied rigorous control filters (Methods) and our binding data are highly reproducible^17^. The FLAG-tagged TF overexpression and reference conditions that we employed could be sources of artifactual binding; however, ChIP under physiological conditions with native antibodies also yield similar binding profiles^14^,^17^. Further, we have shown that our TF overexpression levels are less than or equal to TF gene expression changes in publically available array studies for over 80% of TFs (Supplementary Fig. 4b and ref. 24).

Another possibility is that most previous studies, which assess protein–DNA interactions at specific candidate sites, may consistently underestimate the actual extent of binding. Since early groundbreaking work with the Lac operon^47^, researchers interested in transcriptional control have focused on individual gene expression changes and thus may have systematically understudied the possibility of transcriptionally dormant binding. In fact, the most common approach to determine TF regulatory targets is transcriptional profiling of a gene disruption mutant, which by definition precludes identification of such binding events. Widespread dormant binding could thus be a phenomenon specific to MTB; however, several recent studies in both eukaryotes and prokaryotes used global approaches and reported unexpectedly widespread binding^14^,^16^,^48^,^49^,^50^, including one study in *E. coli* that was not based on ChIP^43^. In addition, various effects of dormant binding have been reported, including association with chromosome organization, replication and cell division^33^,^43^, altering response kinetics and dynamics at regulation-active loci through transcription factor titration and buffering against noisy input^51^,^52^,^53^, suggesting multiple functional contexts for this phenomenon. These observations, in eukaryotes and archaea as well as bacteria, raise the possibility that widespread dormant binding is a common feature of transcriptional systems everywhere that should be considered when developing gene regulatory networks. The implications of these phenomena for MTB biology and for transcriptional control more broadly are largely unexplored, and warrant additional investigation.

## Methods

### Construction of expression vectors and strains

Our in-house analysis indicated that there are 214 putative DNA-binding genes in the *M. tuberculosis* genome. At the outset of this project we had at our disposal a Gateway Entry Clone library of ~2,600 *M. tuberculosis* open reading frames in the backbone of pDONR221 (PFGRC/Colorado State University under NIAID contract HHSN266200400091c). In the event that a putative DNA-binding gene-of-interest was not included in the extant Entry Clone library, we created entry clones through PCR amplification of the relevant gene template from H37Rv gDNA, adding the necessary Gateway recombination sequences to the PCR product. In total, nine genes proved refractory to subcloning efforts, and so were triaged from subsequent analyses. Including those genes from the PFGRC entry clone library and our subcloning efforts the final putative DNA-binding clone library contains 206 genes. We inserted each of these genes in to an *E. coli*-mycobacterial episomal shuttle vector modified to contain an anhydrotetracycline (ATc)-inducible promoter^54^ and a Gateway cloning recombination cassette (kind gift of Eric Rubin). We further modified this vector to contain an N- or C-terminal FLAG epitope tag—amino-acid sequence: n-DYKDDDDK-c. For the present work the C-terminal FLAG-tagged version was used for all DNA-binding experiments, with the exception of experiments utilizing Rv3133c/DosR, which contained the N-terminal FLAG tag. *M. tuberculosis* H37Rv strains containing these ATc-inducible, FLAG-tagged, expression vectors are available from BEI resources (nr-46512, www.beiresources.org).

### Culturing conditions

*M. tuberculosis* strain H37Rv was cultured in Middlebrook 7H9 with the ADC supplement (Difco) and 0.05% Tween80 at 37 °C with constant agitation. For transformation with ATc-inducible expression vectors and subsequent expansion/experimentation, cultures were grown with the addition of 50 μg ml^−^ hygromycin B to maintain the plasmid. All experiments were performed under aerobic conditions and growth was monitored by OD600. At an OD600 of 0.35, expression of a gene of interest was induced for the approximate duration of one cell doubling (18 h) using an ATc concentration 100 ng ml^−1^ culture.

### Chromatin immunoprecipitation

DNA–protein interactions were characterized by cross-linking 50 ml of culture with 1% formaldehyde while agitating cultures at room temperature for 30 min. Crosslinking was quenched by the addition of glycine to a final concentration of 250 mM. Cells were pelleted, washed in 1 × PBS+1 × protease inhibitor cocktail (Sigma) and resuspended in ChIP Buffer 1 (20 mM KHEPES—pH 7.9, 50 mM KCl, 0.5 mM dithiothreitol and 10% glycerol)+1 × protease inhibitor cocktail. Owing to the thick cell wall of *M. tuberculosis*, samples were mechanically lysed using Lysing Matrix B tubes and three rounds of bead beating at maximum speed for 30 s, with cooling on ice between treatments. Samples were centrifuged for 1 min at 13.2 × *g* to pellet beads. Supernatants were collected and sample volumes were normalized to 500 μl in ChIP Buffer 1. We then utilized a Covaris S2 ultrasonicator at settings: amplitude=20%, power=5, cycles per burst=200, for 16 min to shear chromatin to a uniform size centred around 200 bp. Following shearing, the sample was adjusted to buffer IPP150 (10 mM Tris-HCl—pH 8.0, 150 mM NaCl and 0.1% NP40) and immunoprecipitation of FLAG-tagged proteins was initiated by incubating samples overnight rotating at 4 °C with 10 μg (1:55 dilution) M2 anti-FLAG antibody (Sigma, F1804). The following day, samples were incubated with protein G-coupled agarose beads (Pierce) rotating for 30 min at 4 °C and 90 min at room temperature. Agarose bead-protein complexes were pelleted by centrifugation for 2 min at 2,000 × *g* at which point the supernatant was discarded, and the samples were subjected to five rounds of washing in IPP150 buffer (rotate for 2 min, pellet bead–protein complex, discard supernatant). Increasing the stringency, the final two washes were carried out with TE, pH 8.0. Protein complexes were eluted off the beads in two steps. In the first step, protein–bead complexes were incubated in elution buffer 1 (50 mM Tris-HCl—pH 8.0, 10 mM EDTA and 1% SDS) for 15 min at 65 °C. After pelleting and saving the supernatant, protein–bead complexes were treated with TE—pH 8.0 and 1% SDS for 5 min at 65 °C. Elution supernatants were pooled and the proteins were digested/crosslinks were reversed by incubation with 1 mg ml^−1^ Pronase for 2 h at 42 °C followed by 9 h at 65 °C. Immunoprecipitated DNA was subsequently column-purified using QiaQuick PCR purification columns (Qiagen) and eluted twice with 20 μl 10 mM Tris-HCl, pH 8.5.

### ChIP-seq peak control data set

To determine significance thresholds for peak inclusion in our data set, we generated a ChIP-seq control compendium consisting of 10 different sequencing data sets. As no single control type captures all known or potential ChIP artifacts or biases, we included an array of control types, including the following: WT H37Rv chromatin immunoprecipitated with and without anti-FLAG antibody, chromatin samples from uninduced expression-vector-bearing cells immunopreciptated with and without anti-FLAG antibody as well as chromatin samples from induced non-TF genes immunoprecipitated with anti-FLAG antibody.

### Illumina library prep sequencing

All libraries were prepared according to the standard Illumina protocols. Samples were sequenced on the Illumina GAIIx sequencer, generating unpaired 30–50 million 40-bp reads per sample.

### Read alignment and peak calling

Peak calling was carried out using an in-house algorithm outlined in Supplementary Fig. 10 and available for download at http://networks.systemsbiology.net/mtb. Short reads were aligned to the H37Rv reference genome using Bowtie 0.12.7 with default parameters, resulting in 98% of reads being successfully aligned. Read pileups were converted to wiggle tracks for forward, reverse and cumulative strands, and then searched for local extrema. We then estimated half width at half height for each local maximum (a *de facto* ‘peak’), and using nonlinear least squares optimization we found the optimal Gaussian or Gumbel model distribution that best fit the aligned reads. We assigned 0–1 scores based on relative height, width and drift from starting local maximum of each fitted peak. We then merged the forward, reverse and cumulative results in to a ‘combo peak’ and re-scored that triplet with the addition of score values for separation and relative heights of the forward and reverse strand peak centrepoints. The final score for a single ChIP peak was the product of [ScoreF * ScoreR * ScoreC * Sep * EqHts] on a 0–1 scale, with 1 being a ‘perfect’ score.

### Assigning significance scores to called peaks

Each sequencing data set was subjected to the ‘read alignment and peak calling’ algorithm. To determine significance scores of experimental data, we collapsed the peaks (with respective scores) from the 10 control experiments described above into a single data set containing 2,027 scored ‘final’ peaks as negative controls (Supplementary Table 5). For each scored peak in an experimental data set we measured the probability of identifying a comparably high-scoring matched peak type in the control data set. Thus, an experimental peak with *P*=0 indicates that no peaks in the negative control set had an equivalently robust score. Similarly, a peak with a *P* value of 0.01 has a peak quality score better than 99% of all peaks identified in the negative control set. A table with scoring metrics and significance scores for all DNA-binding proteins assayed, all peaks, is provided in Supplementary Table 1.

### RNA isolation

RNA was isolated as described previously^55^. Briefly, cell pellets in Trizol were transferred to a tube containing Lysing Matrix B (QBiogene Inc.) and vigorously shaken at maximum speed for 30 s in a FastPrep 120 homogenizer (Qbiogene) three times, with cooling on ice between steps. This mixture was centrifuged at maximum speed for 1 min and the supernatant was transferred to a tube containing 300 μl chloroform and Heavy Phase Lock Gel (Eppendorf North America Inc.), inverted for 2 min and centrifuged at maximum speed for 5 min. RNA in the aqueous phase was then precipitated with 300 μl isopropanol and 300 μl high-salt solution (0.8 M Na citrate, 1.2 M NaCl). RNA was purified using an RNeasy kit following the manufacturer’s recommendations (Qiagen) with one on-column DNase treatment (Qiagen). Total RNA yield was quantified using a Nanodrop (Thermo Scientific).

### Microarray analysis

RNA was converted to Cy dye-labelled cDNA probes as described previously^27^. Briefly, for all microarrays described here, 3 μg of total RNA was used to generate probes. Sets of fluorescent probes were then hybridized to custom NimbleGen tiling arrays consisting of 135,000 probes spaced at ~100-bp intervals around the *M. tuberculosis* H37Rv genome (NCBI Geo Accession no.: GPL14896). Arrays were scanned and spots were quantified using Genepix 4000B scanner with the GenePix 6.0 software. These data were exported to NimbleScan for mask alignment and robust multichip average normalization^56^. Subsequent statistical analysis and data visualization were carried out using the Arraystar software. To compare against a standard, baseline, expression set, median expression values were calculated for all genes across all input microarrays (*N*=702). Altered gene expression was considered significant if it produced an empirical Bayes method *P*<0.01. Raw microarray data are available at the gene expression omnibus in series GSE59086. Additional details can be found in ref. 24.

### Promoter window size analysis

Receiver operation curves (ROCs) were used for assessing the accuracy of promoter window sizes to associate binding with target regulation. Upstream promoter window sizes were tested every 10 nucleotides from −10 to −200 upstream of designated start sites and at varying nucleotide lengths to −1,500 upstream. Similarly, window sizes were tested every 10 nucleotides from +10 to +200 downstream. The set of ChIP-seq binding events with target regulation was formed by instances within a given window size that a particular TF has a significant overlap of proximal gene targets and differentially expressed genes (as determined in ref. 24). The overlap was computed using hypergeometric enrichment *P* values. The ROC curves were formed by considering the overlap of each possible pairwise combination of TFs and measuring the sensitivity and specificity of the overlap, where sensitivity represents the fraction of differentially expressed target genes that had a binding peak within the promoter window, and specificity represents the fraction of nondifferentially expressed target genes that did not have a binding peak within the promoter window. The R open-source package pROC was used to calculate area under the curve values of tests performed at each window size^57^. The optimal window size was determined by the largest AUC in the upstream and downstream regions and resulted in a −150:+70 window. As a result, genes targeted by a particular TF were identified by having a significant ChIP-Seq-binding peak in the −150:+70 window of their start site or by being part of an operon with a binding site in the −150:+70 region of an upstream gene in the operon.

### Identifying consensus motifs from ChIP-seq data

For consensus motif determination we searched for conserved DNA signatures within ±50 nucleotides of ChIP-seq peak centres using MEME^36^. The peaks were further subdivided into subsets that were only within or outside of our defined promoter windows (thus, there are three subsets for each TF—‘all’ significant peaks, those ‘in’ promoters and those ‘out’). We performed each motif search twice for each grouping—one unconstrained and one constrained to detect only palindromes. For each motif, we computed the significance of the distribution of its locations relative to the corresponding peak centres, relative to a uniform null distribution, using the Komolgorov–Smirnov test. We also scanned each motif in an unbiased manner across the entire genome using FIMO^58^ and computed whether these scanned locations were significantly located within ±50 nt of the corresponding ChIP-seq peak locations relative to randomly sampled locations throughout the genome. Motifs with MEME *E*-values (E <=1) and peak location (*P*<=0.05) were considered significant.

### Recombinant Rv0494 protein purification

The Rv0494 CDS was subcloned into the pET28b expression vector (Novagen/EMD Millipore). The Rv0494 locus was PCR-amplified from purified H37Rv gDNA, adding an XhoI restriction endonuclease site to the 3′ end of the cassette. The primer specific to the 5′ end of the gene cassette contained an NdeI RE site as well as the recognition motif for the HRV 3C protease. After ligation, pET28-Rv0494Ab inducibly expressed Rv0494 with an N-terminal 6x-HIS tag upstream of the HRV 3C cut site and native Rv0494 sequence. For recombinant protein production we transformed BL21(DE3) *E. coli* with pET28-Rv0494Ab. Cultures were grown to an OD600 of 0.5 in Terrific Broth before treatment with 100 μM isopropyl-β-D-thiogalactoside shaking overnight at 18 °C. Following sonication, recombinant protein was recovered from crude lysates by Fast Protein Liquid Chromatography (FPLC) metal affinity chromatography and size exclusion chromatography. To remove the 6x-HIS tag from the final protein product, recombinant Rv0494 was subjected to HRV 3C protease (Novagen) digestion (rotating overnight at 4 °C). Following cleavage, Rv0494 solutions were again passed over a metal affinity column to remove the liberated epitope tag and the HIS-tagged protease. Final purification was effected through size exclusion chromatography. Protein aliquots were snap-frozen in storage buffer (150 mM NaCl, 20 mM Tris-HCl—pH 7.5, 5% glycerol) and kept at −80 °C for subsequent applications. The purified 26-kDa Rv0494 protein contains two non-native amino acids at the N terminus.

### Universal electrophoretic mobility shift assays

Similar to the technique described in ref. 42, for uEMSAs, three oligos (Integrated DNA Technologies) were resuspended to 50 μM in dsDNA annealing buffer (10 mM Tris-HCl—pH 7.5, 100 mM NaCl, 1 mM EDTA). In this scheme, oligo 1 consisted of 30 nucleotides taken directly from the Rv3094c–Rv3095 intergenic region in the *M. tuberculosis* genome, or the consensus motif sequences flanked by GC-matched randomized nucleotides. Oligo 2 consisted of 42 nucleotides: the reverse complement of the oligo 1 30-mer, as well as a 12-nucleotide ‘scaffold’ sequence at the 3′ end to which oligo 3 is the reverse complement. Oligo 3 consisted of a 12-mer with a IR680 or IR800 infrared dye covalently coupled to the 5′ end. The IR680 12-mer scaffold/universal sequences were different than the IR800 12-mer. The three ssDNA oligos were combined to a final concentration of 50 μM, vigorously agitated and heated to 95 °C for 10 min on a benchtop heat block. The entire metal block was subsequently removed from heat and allowed to cool to room temperature over a period of ~3 h protected from light. The resulting dsDNA product became the substrate for subsequent EMSA experiments. Purified recombinant Rv0494 protein was removed from storage buffer (150 mM NaCl, 20 mM Tris-HCl—pH 7.5, 5% glycerol) and exchanged to sterile-filtered reaction buffer (10 mM Tris-HCl—pH 8.0, 10 mM NaCl, 1 mM DTT, 1 mM EDTA, 5 ng μl^−1^ BSA) using a 10-kDa-cutoff spin column (Amicon). In the present study, Rv0494 was present at 0.1 μM for the Rv3094c–Rv3095 and 17-mer consensus motif uEMSA experiments. Rv0494 was present at 2.0 μM for the 9-mer consensus motif uEMSA experiments. Fifty-nanometre specific, IR680-labelled, dsDNA target was used in all reactions. For specific and nonspecific competition experiments, 20 × molar excess IR800-labelled dsDNA was added to the reaction mixture (final concentration=1 μM). All components of a reaction were combined, mixed and incubated protected from light for 30 min at room temperature. Fifteen microlitres of reaction product was loaded on to 10% polyacrylamide TBE gel and run at a constant 150 V for 75 min, protected from light. Owing to the lower melting temperature of the universal 12-mer used in these experiments (~65 °C), the gel box was contained in an ice bath for the duration of electrophoresis. The gel was washed once in PBS before visualization on a Li-cor Odyssey scanner.

## Additional information

**How to cite this article**: Minch, K. J. *et al.* The DNA-binding network of *Mycobacterium tuberculosis*. *Nat. Commun.* 6:5829 doi: 10.1038/ncomms6829 (2015).

## Supplementary Material

Supplementary Figures Supplementary Figures 1-10

Supplementary Data 1 Table of all p<0.01 ChIP-seq peaks for all transcription factors in this study.

Supplementary Data 2 Table of the inter-/intragenic distribution of the highest scoring 20% of all ChIP peaks presented on a TF-by-TF basis.

Supplementary Data 3 Table of all binding events in -150 to + 70 nucleotide promoter window.

Supplementary Data 4 Table of the two most significant DNA consensus motifs identified for every transcription factor in this study.

Supplementary Data 5 Table of all peaks with associated peak scores for the "control peak" dataset
