# Supplementary material for: The DNA-binding network of Mycobacterium tuberculosis
Source: Nat Commun. 2015 Jan 12;6:5829. doi: 10.1038/ncomms6829 (PMC4301838; doi:10.1038/ncomms6829)
Supplement: Supplementary Figures — 1-10 [file ncomms6829-s1.pdf]

Supplementary Figure 1

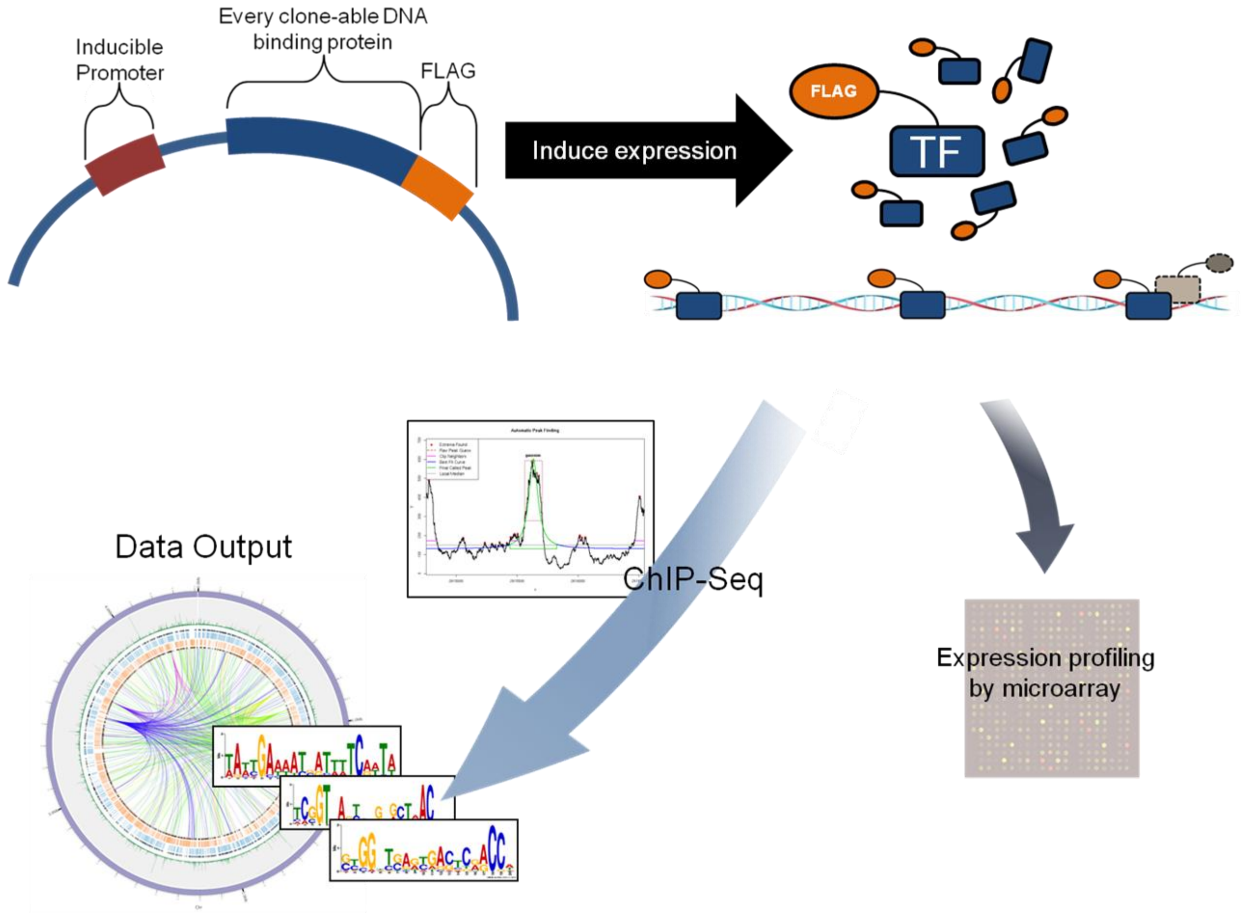

Supplementary Figure 1 – Overview of experimental system and workflow in this study. 206 out of 214 TFs encoded in the H37Rv genome were cloned into an epitope-tagged anhydrotetracycline (ATc)-inducible episomal plasmid and transformed into MTB. Expression of the gene-of-interest was induced with 100ng/ml ATc for one MTB cell division period (18 hours),at which point samples were collected and prepared for analysis by expression profiling by microarray (see [24] for details) or by ChIP-seq. Sequencing read alignment and ChIP peak identification as described in the Materials and Methods preceded network filtering and downstream data analysis.

Supplementary Figure 2

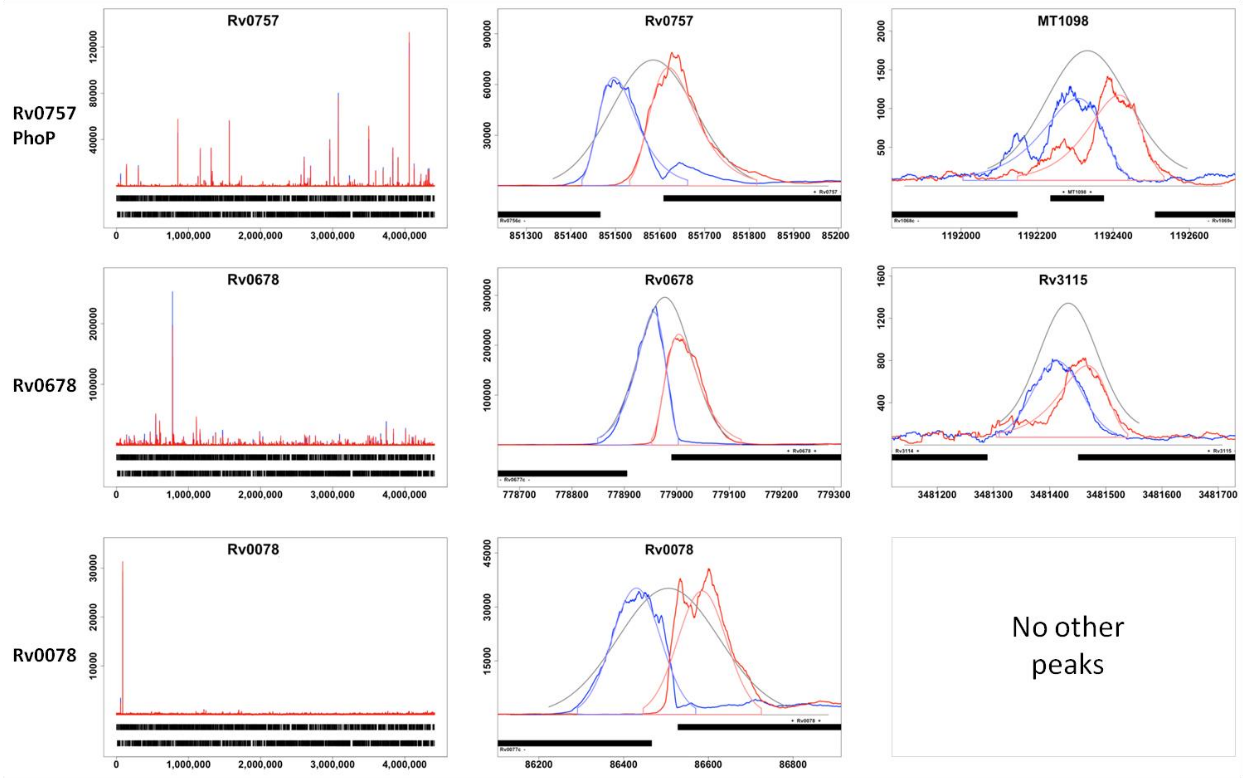

Supplementary Figure 2 – Characteristics of global and local ChIP binding data. For each ChIP experiment we aligned reads to the reference genome and called peaks. **Left Panels**: For each TF all ChIP-seq reads were aligned to the MTB H37Rv reference genome (x-axis = 4.4 Mb, Y-axis scales vary by sequencing read depth and peak enrichment). **Middle Panels:** The most significant peaks, those with p <0.001 as compared to the control data set, exhibit substantial enrichment over background read-depth, bimodal distribution of forward and reverse strand reads, a relatively uniform Gaussian or Gumbel distribution of reads, and height-width proportional ratios on each strand. **Right Panels:** Peaks with lower significance scores – those binding sites with p values of ~0.01 – tend to be less enriched over background read-depth, have read pileups/peak shapes that deviate farther from ideal distributions, and demonstrate greater variance in height-to-width ratios. These deviations from optimal peak shape become progressively more pronounced for ChIP peaks with lower confidence scores. Importantly, these peak regions can still be readily differentiated from local background and in some cases we have confirmed binding at these sites by independent means (see Fig. 4 of main text); however, their cumulative score demerits make them more similar to peaks identified in the negative control set. For middle and right column panels the blue and red lines are forward and reverse strand alignments, respectively. The grey lines indicate the best fit distribution of the forward and reverse strand alignments.

Supplementary Figure 3

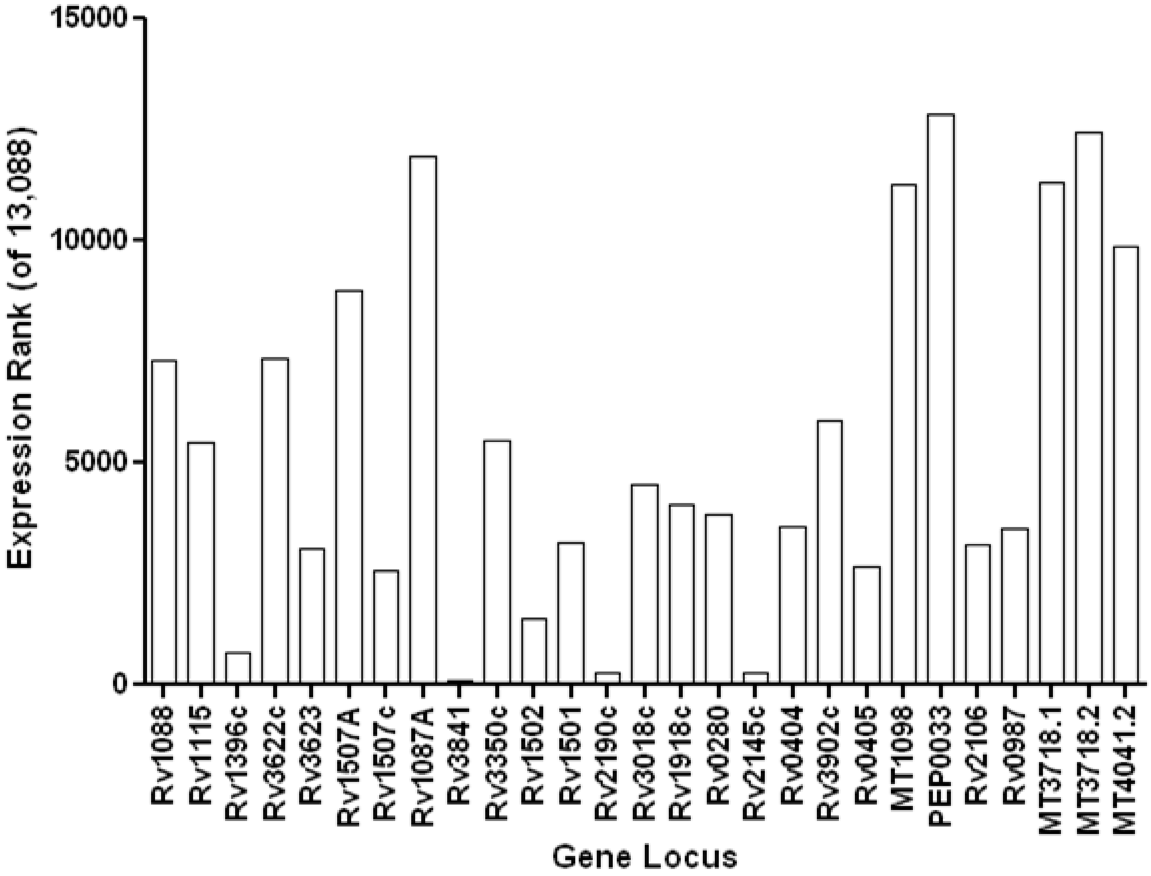

Supplementary Figure 3 – Expression rank of 20 most-bound DNA regions. In accordance with recent literature (see main text) we compared the RNA expression levels of genes proximal to the most-bound DNA regions of the MTB chromosome as determined in this study. Ordered from left to right these are the genomic loci bound by the most TFs (Rv1088 = 134 TFs, Rv0405 = 41 TFs) Reported is the rank of the median absolute expression level of each feature derived from 702 microarray experiments (see [24]).

Supplementary Figure 4

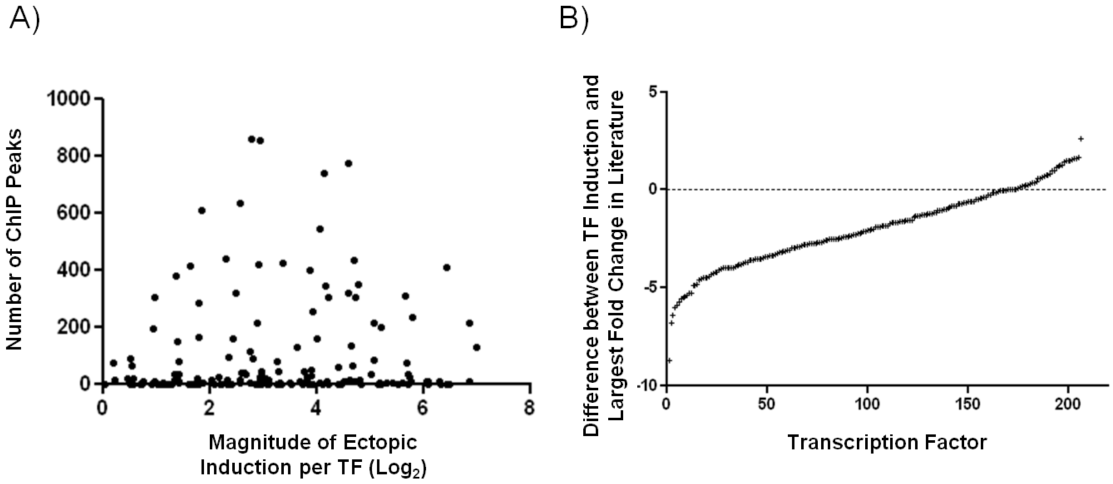

Supplementary Figure 4 – Magnitude of TF ectopic induction. A) The ectopic induction level of each TF reported in this study was determined against the median level of that TF derived from 702 microarray experiments[24]. Shown is the TF ectopic induction (log_2) vs. the number of ChIP peaks identified for that TF. There is no correlation between magnitude of induction and the number DNA sites (Spearman's rho = 0.029). **B)** For each transcription factor the largest fold change found in a collection of 2,325 publically available expression profiles was identified[6]. That fold change was compared to the fold change induced by addition of ATc to an MTB strain carrying the ectopic/inducible TF. The log_2 difference between the two fold changes (FC_{TFOE} – $FC_{literature}$) is plotted. The dashed line indicates an equal level of induction. The majority of TFs (~80%, values below the dashed line) have previously published fold changes larger than those caused by ATc induction.

Supplementary Figure 5

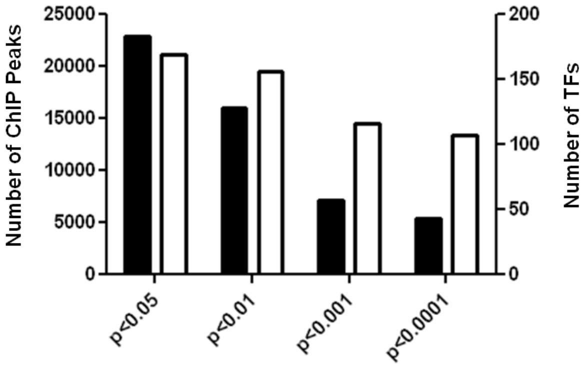

Supplementary Figure 5 – Impact of p-value threshold on the number of binding sites & regulators in network. Increasing stringency results in progressively fewer ChIP peaks (black bars) and transcription factors (white bars) included in final network.

Supplementary Figure 6

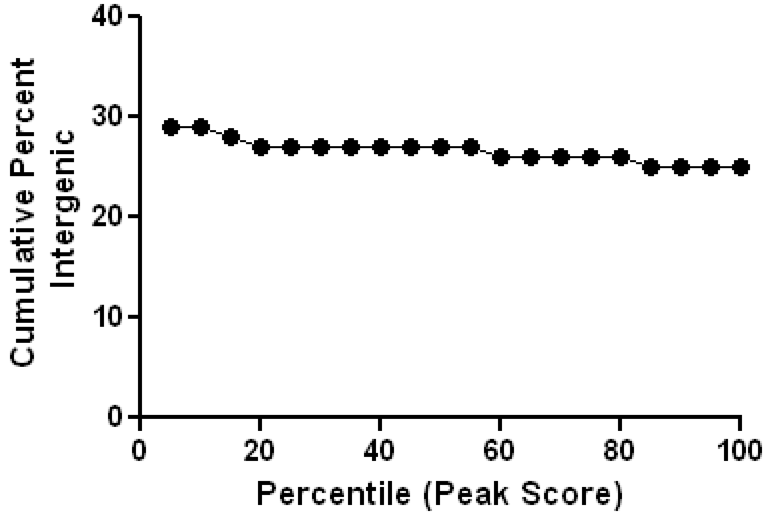

Supplementary Figure 6 – Percent intergenic binding as a function of peak score. ChIP peak score was used to parse all binding data into 5-percentile bins. These bins were analyzed for their binding location relative to annotated CDS boundaries and plotted as cumulative percent intergenic peak location per bin.

Supplementary Figure 7

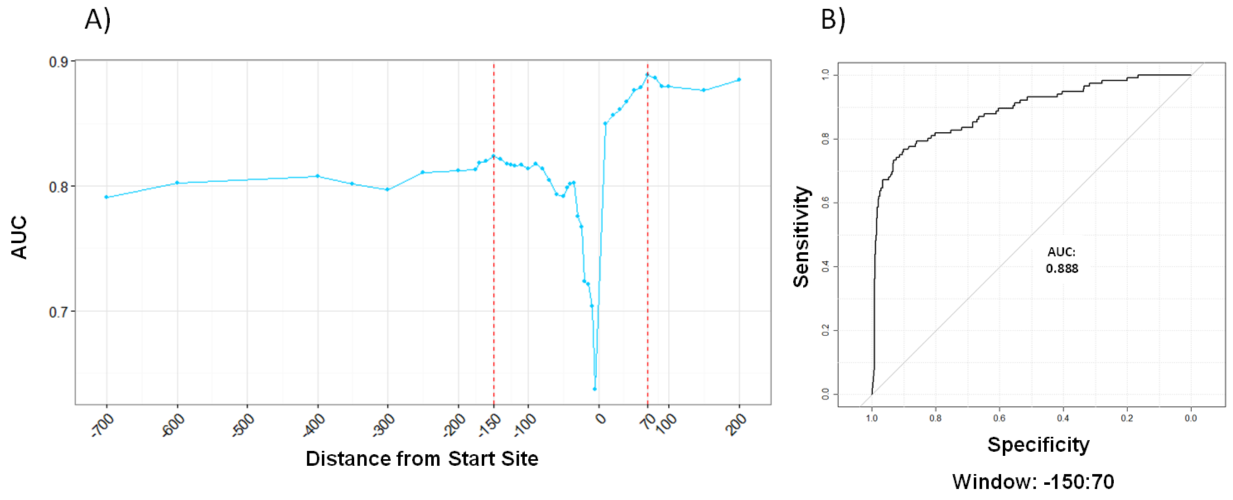

Supplementary Figure 7 – de novo definition of MTB promoter size. A) Upstream promoter window sizes were tested every 10 nucleotides from -10 to -200 upstream of designated start sites and at varying nucleotide lengths to -1500 upstream. Similarly, window sizes were tested every 10 nucleotides from +10 to +200 downstream. The set of ChIP-seq binding events with target regulation was formed by instances within a given window size that a particular TF has a significant overlap of proximal gene targets and differentially expressed genes (as determined in [24]). The overlap was computed using hypergeometric enrichment p-values. The ROC curves were formed by considering the overlap of each possible pairwise combination of TFs and measuring the sensitivity and specificity of the overlap, where sensitivity represents the fraction of differentially expressed target genes that had a binding peak within the promoter window, and specificity represents the fraction of non-differentially expressed target genes that did not have a binding peak within the promoter window. **B)** The R open-source package pROC was used to calculate AUC values of tests performed at each window size. The optimal window size was determined by the largest AUC in the upstream and downstream regions and resulted in a -150:+70 window.

Supplementary Figure 8

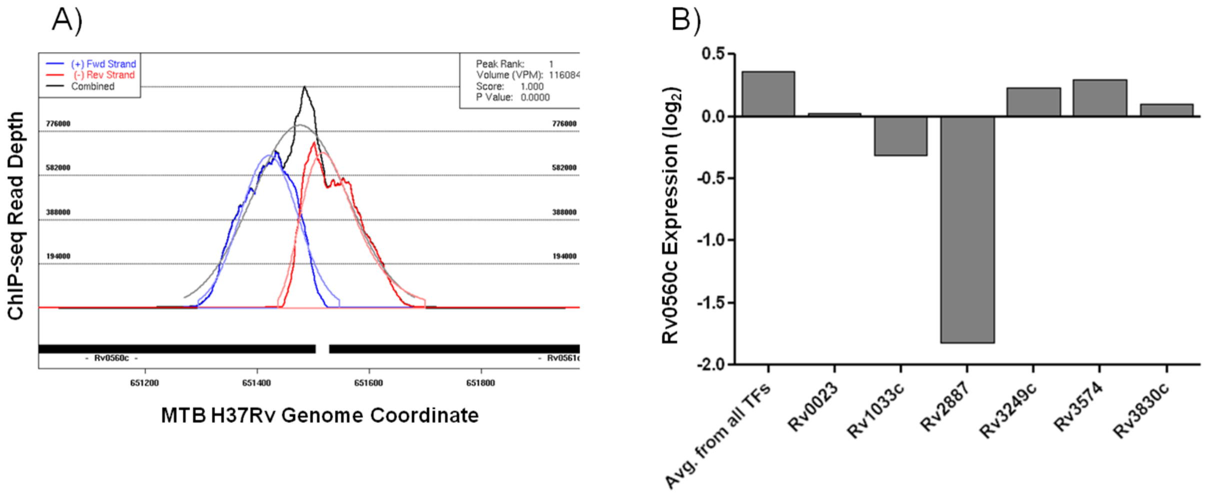

Supplementary Figure 8 – Regulation of Rv0560c. A) ChIP peak demonstrating Rv2887 binding to the promoter region of Rv0560c. **B)** Ectopic induction of Rv2887 led to a significant 3.5-fold repression of Rv0560c (empirical Bayes method $p < 7.0\times10^{-11}$).

Supplementary Figure 9

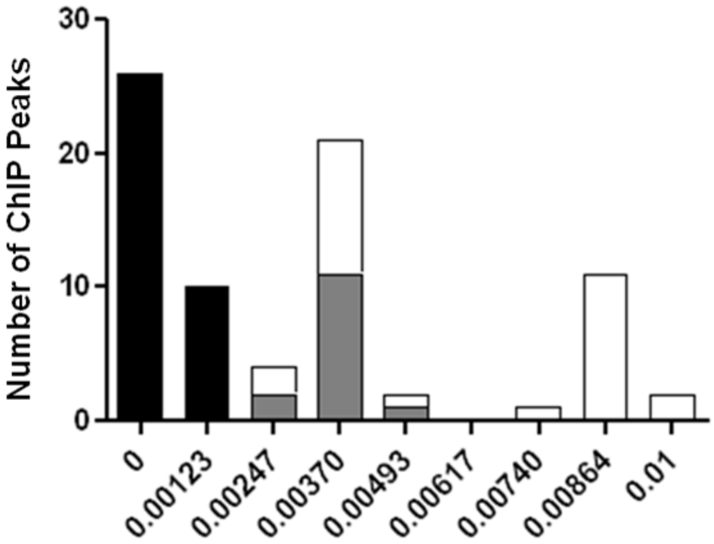

Supplementary Figure 9 – p-value distribution Rv0494 consensus motifs. All Rv0494 binding sites (77 total) were interrogated for consensus motif discovery in iterative searches. The motif corresponding to a palindromic 17-mer was derived from the most-significant ChIP peaks ($p<0.0001$, 36 ChIP peaks, black bars). The second motif identified on the first iteration corresponded to a palindromic 9-mer and was derived from ChIP peaks of $0.0015 < p < 0.01$ (28 ChIP peaks, white bars). DNA sequences identified in the first round of consensus motif discovery were removed, and on a second iteration a close variant of the palindromic 17-mer consensus motif was identified from peaks with mid-range significance values ($0.0015 < p < 0.004$; 13 ChIP peaks, grey bars).

Supplementary Figure 10

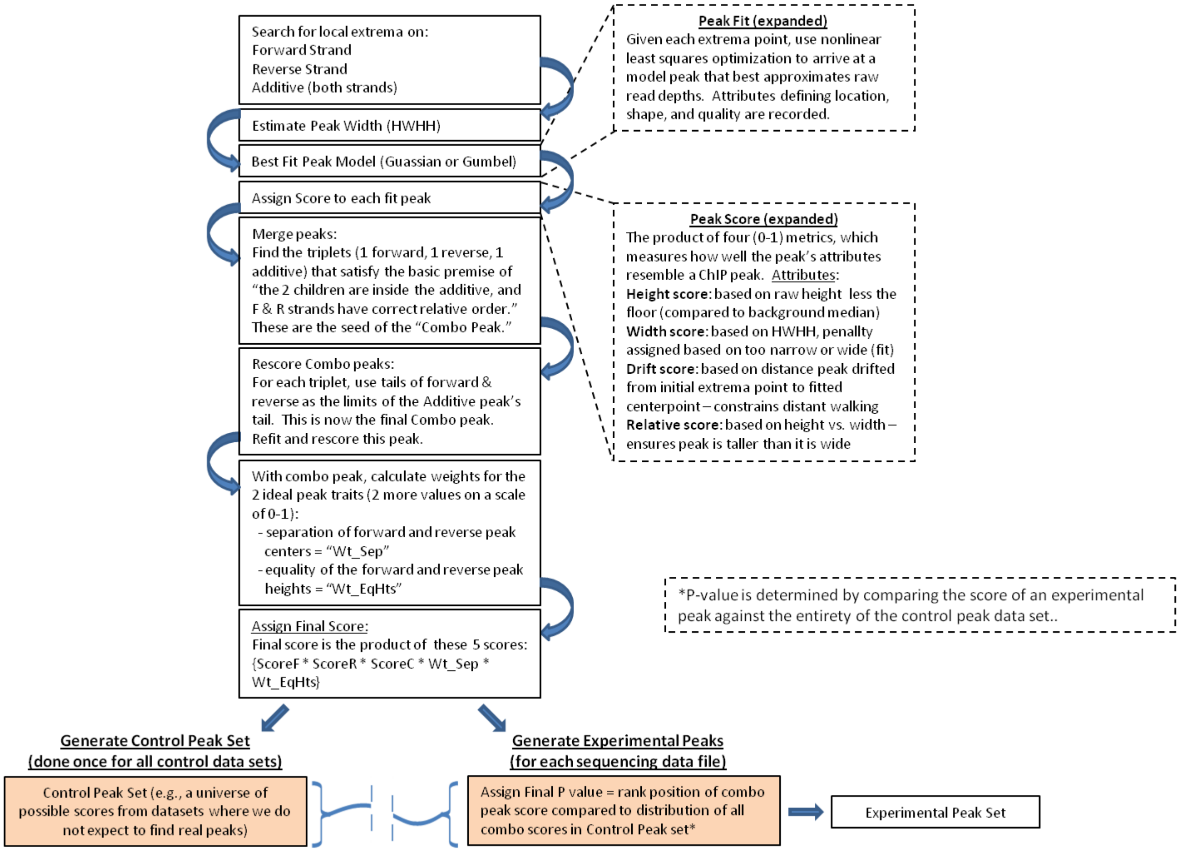

Supplementary Figure 10 – Peak Calling Workflow.
